# CT imaging findings of abdominopelvic vascular compression syndromes: what the radiologist needs to know

**DOI:** 10.1186/s13244-020-00852-z

**Published:** 2020-03-17

**Authors:** Cecilia Gozzo, Dario Giambelluca, Roberto Cannella, Giovanni Caruana, Agita Jukna, Dario Picone, Massimo Midiri, Giuseppe Salvaggio

**Affiliations:** 1 Sezione di Scienze Radiologiche, Biomedicina, Neuroscienze e Diagnostica avanzata (BIND), University of Palermo, Via del Vespro 129, 90127 Palermo, Italy; 2 Department of Medical Surgical Sciences and Advanced Technologies “G.F. Ingrassia”-Radiology I Unit, University Hospital “Policlinico Vittorio Emanuele”, Catania, Italy; 3 Radiology Research Laboratory, Riga Stradins University, Riga, Latvia; 4 Department of Pathology, Riga Stradins University, Riga, Latvia

**Keywords:** Computed tomography, Abdomen, Vascular compression syndrome, Vascular syndromes

## Abstract

Abdominopelvic vascular compression syndromes include a variety of uncommon conditions characterized by either extrinsic compression of blood vessels by adjacent anatomical structures (i.e., median arcuate ligament syndrome, nutcracker syndrome, May-Thurner syndrome) or compression of hollow viscera by adjacent vessels (i.e., superior mesenteric artery syndrome, ureteropelvic junction obstruction, ureteral vascular compression syndromes, portal biliopathy). These syndromes can be unexpectedly diagnosed even in asymptomatic patients and the predisposing anatomic conditions can be incidentally discovered on imaging examinations performed for other indications, or they can manifest with atypical abdominal symptoms and acute complications, which may lead to significant morbidity if unrecognized. Although computed tomography (CT) is an accurate noninvasive technique for their detection, the diagnosis remains challenging due to the uncommon clinical presentation and often overlooked imaging features. Dynamic imaging may be performed in order to evaluate patients with inconstant symptoms manifesting in a specific position. The purposes of this paper are to review the CT imaging findings of abdominopelvic vascular compression syndromes, correlating with anatomical variants and to provide key features for the noninvasive imaging diagnosis.

## Key points:

- Abdominopelvic vascular compression syndromes present with a wide spectrum of clinical manifestations.
- Contrast-enhanced CT allows the identification of typical imaging features and possible complications.
- Predisposing anatomical conditions are encountered on CT examinations performed for unrelated indications.

## Introduction

Abdominopelvic vascular compression syndromes include a large spectrum of abdominal conditions (Table 1) characterized by either extrinsic compression of blood vessels by adjacent anatomical structures (i.e., median arcuate ligament syndrome, nutcracker syndrome, May-Thurner syndrome) or compression of hollow viscera by adjacent vessels (i.e., superior mesenteric artery syndrome, ureteropelvic junction obstruction, ureteral vascular compression syndromes, portal biliopathy) [1]. The exact prevalence of these syndromes is not known, but it can be estimated based on the prevalence of predisposing anatomical variants. Clinically, these syndromes are typically encountered in young and otherwise healthy patients and may manifest with a variety of nonspecific abdominal symptoms, ranging from vague abdominal pain, nausea and vomiting, loss of weight to acute abdominal hemorrhage, arterial ischemia and embolism or venous stasis and thrombosis, making the clinical diagnosis particularly challenging [2]. The predisposing anatomic abnormalities may be incidentally discovered even in asymptomatic patients on abdominal imaging examinations performed for unrelated indications [1]. Their recognition in asymptomatic patients is relevant for preventing vascular injuries during surgical procedures or for prompt treatment of possible complications related to these syndromes (i.e., aneurysm rupture in median arcuate ligament syndrome, deep venous thrombosis and pulmonary embolism in May-Thurner syndrome or urinary tract infections in ureteropelvic junction obstruction).

**Table 1:** Abdominopelvic vascular compression syndromes

Compression syndrome	Cause	Clinical features	CT key findings	Treatment
**Median arcuate ligament syndrome**	Compression of the celiac artery by the median arcuate ligament	Chronic postprandial epigastric pain, nausea and loss of weight	The hooked appearance of the celiac artery in the absence of atherosclerotic plaques; post-stenotic dilatation; collateral vessels; true pancreaticoduodenal arteries aneurysms	**Endovascular:** embolization of pancreaticoduodenal aneurysms and celiac artery revascularization with stent placement; **surgical:** transection of the median arcuate ligament, celiac ganglionectomy and bypass surgery
**Nutcracker syndrome**	**Anterior NCS:** compression of the LRV between the aorta and the SMA	Hematuria, gonadal vein reflux, and pelvic varices	“Beak sign” of LRV; AMA lower than 35°; AMD from 2 to 8 mm	**Conservative:** in patients with tolerable symptoms. **Surgical for anterior NCS:** transposition of LRV or left gonadal vein, saphenous vein cuff or vein patch; **for posterior NCS:** anterior reimplantation of retroaortic LRV; **endovascular:** LRV stenting for the treatment of NCS associated with pelvic congestion
	**Posterior NCS:** compression of the LRV between the aorta and vertebral body			
**May-Thurner syndrome**	Compression of the left common iliac vein between the overlying right common iliac artery and the V lumbar vertebra	Left lower extremity swelling, edema, varicose veins, venous ulcers, acute pulmonary embolism or phlegmasia cerulea dolens	Iliac vein compression and adjacent deep vein thrombosis	**Conservative:** in absence of deep venous thrombosis. **Endovascular:** stent placement with thrombolysis or anticoagulation therapy in patients with acute venous thrombosis; **surgical:** thrombectomy, vascular transposition, venous bypass, and venoplasty
**Superior mesenteric artery syndrome**	Compression of the third portion of the duodenum between the abdominal aorta and the SMA	Postprandial abdominal pain, loss of weight, nausea, and vomiting	Compression of the third portion of the duodenum, with upstream severe dilatation of proximal duodenum and stomach; AMA lower than 22°; AMD shorter than 8 mm	**Conservative:** decompression through nasogastric tube placement; **surgical:** duodeno-jejunostomy for patients with severe symptoms
**Ureteropelvic junction obstruction**	Compression of the ureteropelvic junction by “crossing vessels” (i.e. lower pole segmental renal vessels)	Flank pain, hematuria, urolithiasis, urinary tract infections or pyelonephritis	Hydronephrosis; renal pelvis with inverted “teardrop” appearance, which typically “drapes” over the lower pole segmental vessel	**Surgical:** endopyelotomy, pyeloplasty and vessel transposition
**Ureteral vascular compression syndromes**	Compression of the ureter by adjacent common iliac artery aneurysm or dilated or aberrant ovarian vein	Flank pain, hematuria or pyelonephritis	Hydronephrosis and ureter dilatation by common iliac artery aneurysm or a dilated ovarian vein in absence of urinary calculi or tumoral strictures	**Endovascular:** transcatheter ovarian vein embolization; **surgical:** laparoscopic uretero-ureterostomy, ovarian vein ligation
**Portal biliopathy**	Compression of biliary ducts by “portal cavernoma”	Chronic cholestasis, jaundice, choledocholithiasis, cholangitis, and secondary biliary cirrhosis	Bile duct dilatation; the presence of portal cavernoma; acute angulation of the common bile duct forming a “kinking”, “scalloping” or “wavy” delineation of the extrahepatic biliary ducts	**Interventional:** nasobiliary or biliary stent placement and portal vein recanalization with TIPS placement; **surgical:** hepaticojejunostomy or choledochoduodenostomy

*NCS* Nutcracker syndrome, *LRV* Left renal vein, *SMA* Superior mesenteric artery, *AMA* Aortomesenteric angle, *AMD* Aortomesenteric distance, *OVS* Ovarian vein syndrome, *TIPS* Transjugular intrahepatic portosystemic shunt

The noninvasive diagnosis of abdominopelvic vascular compression syndromes may be unexpectedly made with different imaging modalities including ultrasound, computed tomography (CT), and magnetic resonance imaging (MRI). Among them, contrast-enhanced CT is the most frequently recommended imaging method in clinically suspected syndromes, providing accurate detection of vascular structures and their relationship with adjacent organs [1, 2]. MRI may be preferred in children or young patients due to the absence of ionizing radiation. Dynamic imaging with Doppler ultrasound or CT allows the evaluation of changes in flow velocity or vessel caliber and may be performed in patients with inconstant symptoms [2, 3]. The combination of noninvasive imaging modalities, which allow the precise evaluation of anatomical structures, and invasive techniques, such as venography or arteriography that are useful for the direct measurement of pressure gradients, is helpful to establish the definitive diagnosis. However, the invasive procedures should be limited to the most doubtful cases or when the endovascular treatment needs to be performed.

The purpose of this article is to review the pathophysiological and clinical background of abdominopelvic vascular compression syndromes and the most relevant CT imaging findings for the diagnosis.

## Median arcuate ligament syndrome

**Fig. 1:**
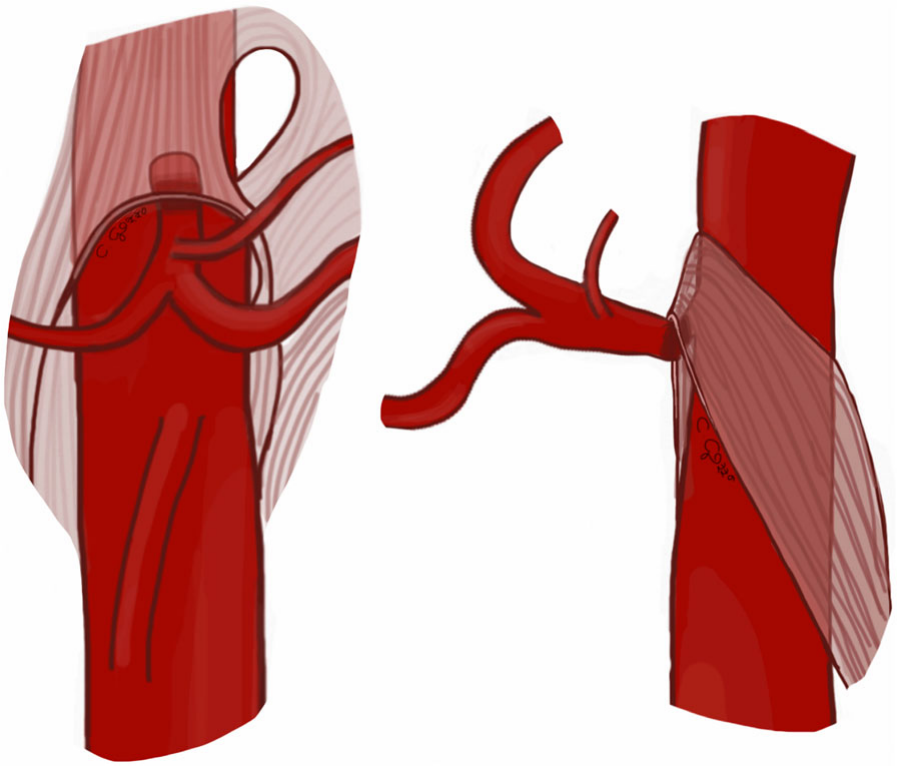
Drawing illustrating the coronal and sagittal views of the median arcuate ligament compressing the root of the celiac artery

Median arcuate ligament syndrome (MALS), also called “Dunbar syndrome”, represents a condition characterized by external compression of celiac artery root by the median arcuate ligament (Fig. 1). This latter is a fibrous arch connecting the right and left diaphragmatic crura on either side of aortic hiatus. Two anatomic conditions can predispose to MALS: the higher origin of the celiac artery or the lower insertion of median arcuate ligament [3]. These anatomical variants are present in about 10–24% of the general population [3, 4]. Overall, MALS is more commonly encountered in young thin women [2, 3].

The classic clinical manifestations of MALS include chronic postprandial epigastric pain, nausea, and loss of weight due to dynamic compression of the celiac artery [1]. However, this anatomical anomaly is asymptomatic in up to 85% of patients and may be incidentally encountered on CT examinations performed for unrelated reasons [1, 5, 6]. The mechanism of pain is still debated. During expiration, the abdominal aorta and its branches are displaced superiorly and the median arcuate ligament compression of the celiac artery increases. This may be responsible for a steal phenomenon with blood flow diverted away from the superior mesenteric artery to the celiac artery branches trough the collateral pathway of pancreaticoduodenal arcades [1, 4]. Epigastric pain may be also related to celiac plexus nerve compression [7]. The development of collateral circulation through the pancreaticoduodenal arcades prevents chronic hypoperfusion in patients with severe stenosis of the celiac artery [6].

**Fig. 2:**
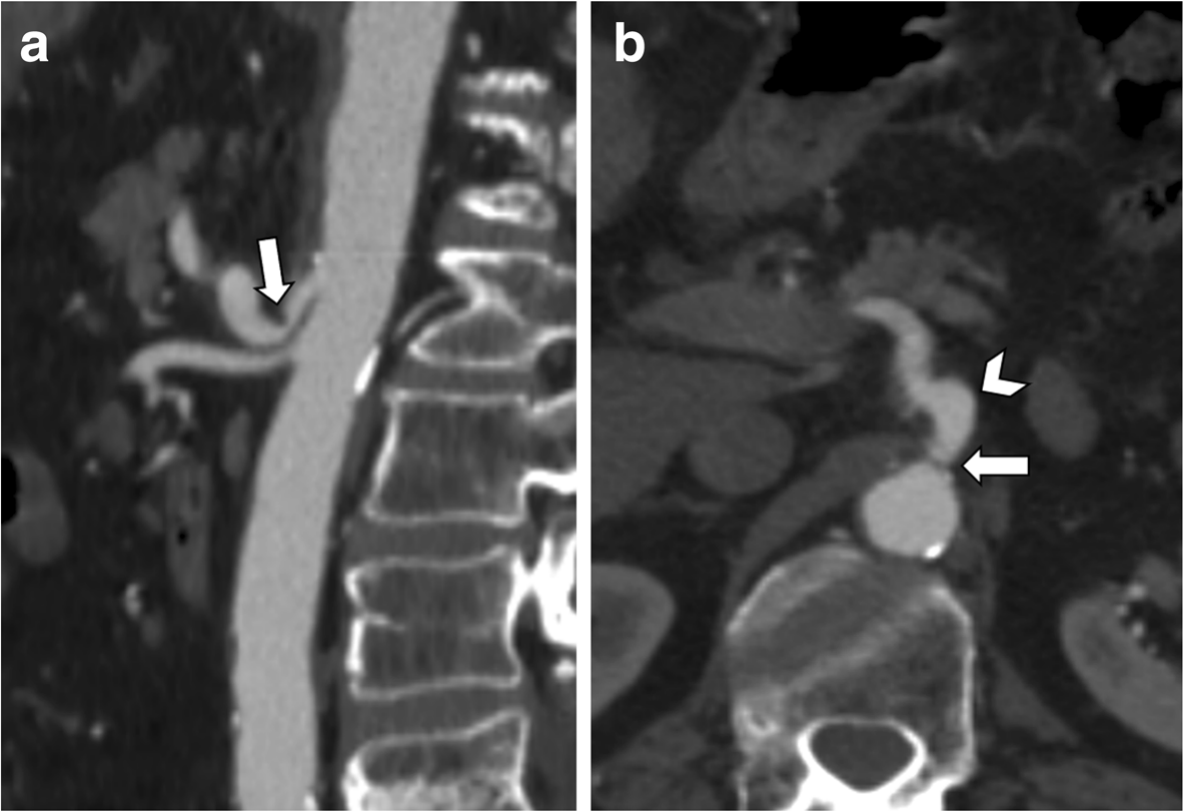
A 65-year-old man with median arcuate ligament compressing the celiac artery. **a** Sagittal CT image shows the “hooked appearance” (arrow) with severe stenosis at the origin of the celiac artery, in the absence of atherosclerotic plaques. **b** Corresponding axial CT image shows celiac artery compression (arrow) with post-stenotic dilatation (arrowhead)

In children and young adults, the Doppler ultrasound examination may directly demonstrate the dynamic variations of flow velocity in the celiac artery during respiratory excursions. Indeed, celiac artery compression at the end of expiration markedly increases the flow velocity [8]. Dynamic CT examination may also be performed in both deep inspiration and expiration in order to evaluate the dynamic modifications in celiac artery diameter [1]. CT imaging should include the early arterial phase acquired in deep expiration in order to increase the proximal celiac trunk compression by the median arcuate ligament, followed by the portal venous phase in deep inspiration [9]. Sagittal and coronal images should be included for optimal visualization of the celiac artery. The proximal narrowing of the celiac trunk can be better depicted on sagittal CT reconstructions, demonstrating a focal indentation on the superior surface of the vessel with a typical “hooked appearance” (Fig. 2), in the absence of atherosclerotic plaques or other causes of extrinsic compression. However, calcified plaques may coexist in older patients and make the differential diagnosis of celiac artery stenosis challenging. In these cases, the lack of hooked appearance on sagittal CT images is the only feature to rule out the presence of MALS [5]. Post-stenotic dilatation and development of peripancreatic collaterals can also be visualized on CT images, and they are imaging features suggesting the proximal compression of the celiac artery. Sugae et al. [10] proposed a grading system of celiac artery stenosis caused by median arcuate ligament compression based on stenosis grade, length of stenosis, distance from the abdominal aorta and presence of small collateral vessels found on CT angiography. Severe stenosis of the celiac artery may be particularly relevant in patients undergoing pancreatic surgery with pancreaticoduodenectomy and it is a significant risk factor for upper abdominal organs infarction [11]. In these patients, the celiac artery remains the only vessel for the arterial blood supply of the upper abdominal organs due to the transection of the pancreaticoduodenal artery during the surgical procedure.

**Fig. 3:**
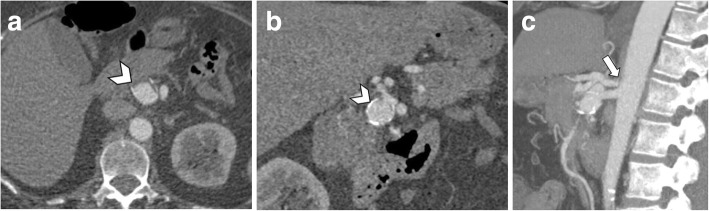
A 63-year-old woman with median arcuate ligament compressing the celiac artery. Axial (**a**) and coronal (**b**) arterial phase CT image shows pancreaticoduodenal artery aneurysm (arrowheads). Reconstructed MIP images on the sagittal plane (**c**) show severe stenosis at the origin of the celiac artery (arrow) with post-stenotic dilatation on arterial phase images acquired during expiration

The most concerning consequence of MALS is the visceral artery aneurysm formation. Severe celiac artery stenosis may lead to altered hemodynamic in the pancreaticoduodenal arcades, causing intimal damage and dysfunction of the medial arterial layer with possible formation of true pancreaticoduodenal arteries aneurysms (Fig. 3). Aneurysm formation should be carefully ruled out as it is correlated with a high possibility of rupture and hemorrhage, developing in up to 40% of patients, with a mortality rate of 50% [3, 4, 12].

The symptomatic MALS may be managed with surgical transection of the median arcuate ligament, celiac ganglionectomy, and bypass surgery, which may provide sustained symptom relief [7]. Endovascular treatments include embolization of pancreaticoduodenal aneurysms and celiac artery revascularization with stent placement prior to surgical decompression [4]. Stent placement alone is contraindicated due to the risk of occlusion caused by sustained extrinsic compression from the median arcuate ligament [4].

## Nutcracker syndrome

**Fig. 4:**
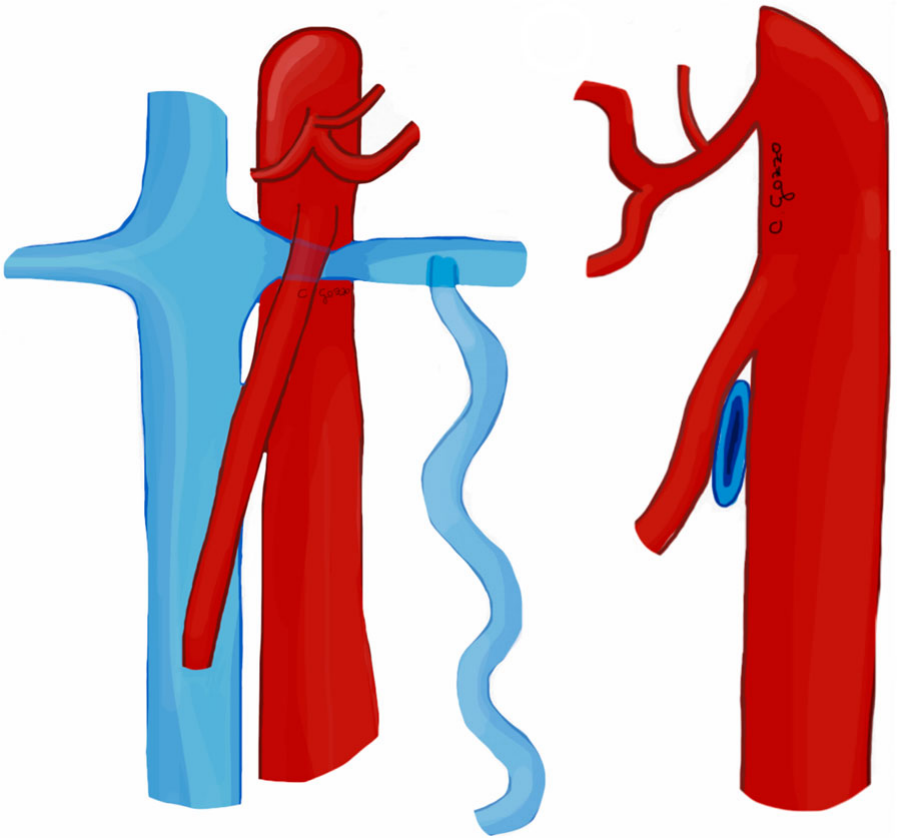
Drawing illustrating the coronal and sagittal views of anterior nutcracker syndrome

**Fig. 5:**
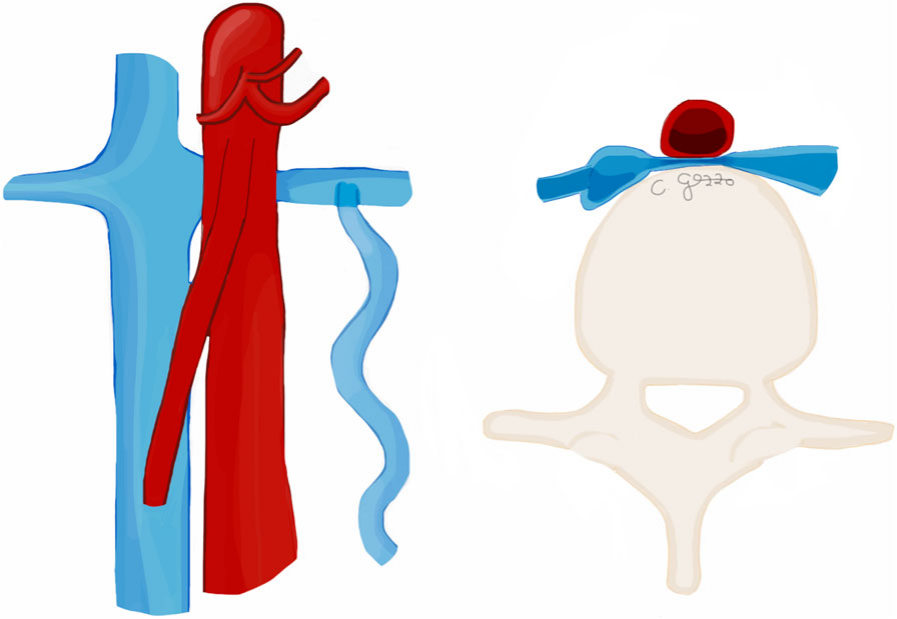
Drawing illustrating the coronal and axial views of posterior nutcracker syndrome

Nutcracker syndrome (NCS) is defined by the compression of the left renal vein (LRV) between the aorta and the superior mesenteric artery (SMA), known as “anterior nutcracker syndrome” (Fig. 4), or more rarely between the aorta and a vertebral body, recognized as “posterior nutcracker” or “pseudo-nutcracker syndrome” (Fig. 5) [13, 14]. The definite prevalence of NCS is unclear due to the high variability in clinical manifestations [15, 16]. Some anatomical variants may cause NCS. The retroaortic LRV, an anatomical variant seen in about 3% of the population, can be responsible for pseudo-nutcracker syndrome in 9% of patients with left-sided varicocele [17]. The “renal collar”, also known as “circumaortic venous ring”, is another anatomic variant characterized by the presence of two LRVs passing anteriorly and posteriorly to the aorta, respectively [17]. In patients with “renal collar” (0.3% to 16% of the population), the concomitant compression of the anterior LRV—between the abdominal aorta and SMA—and the posterior LRV—between the abdominal aorta and spine—is defined as “combined nutcracker syndrome” [15]. Anterior NCS may coexist with SMA syndrome due to their common pathogenesis [1]. Rapid loss of weight and retroperitoneal fat, ptosis of the left kidney, preaortic fibrous tissue, and duodenal interposition have been reported as predisposing factors for NCS [1, 13].

Nutcracker syndrome may lead to increased venous pressure in the left kidney, resulting in rupture of glomerular capillaries with intermittent hematuria, gonadal vein reflux, and formation of pelvic varices [1]. However, when this anatomic compression is only seen on imaging without clinical symptoms, it should be labeled as “nutcracker phenomenon” [1].

**Fig. 6:**
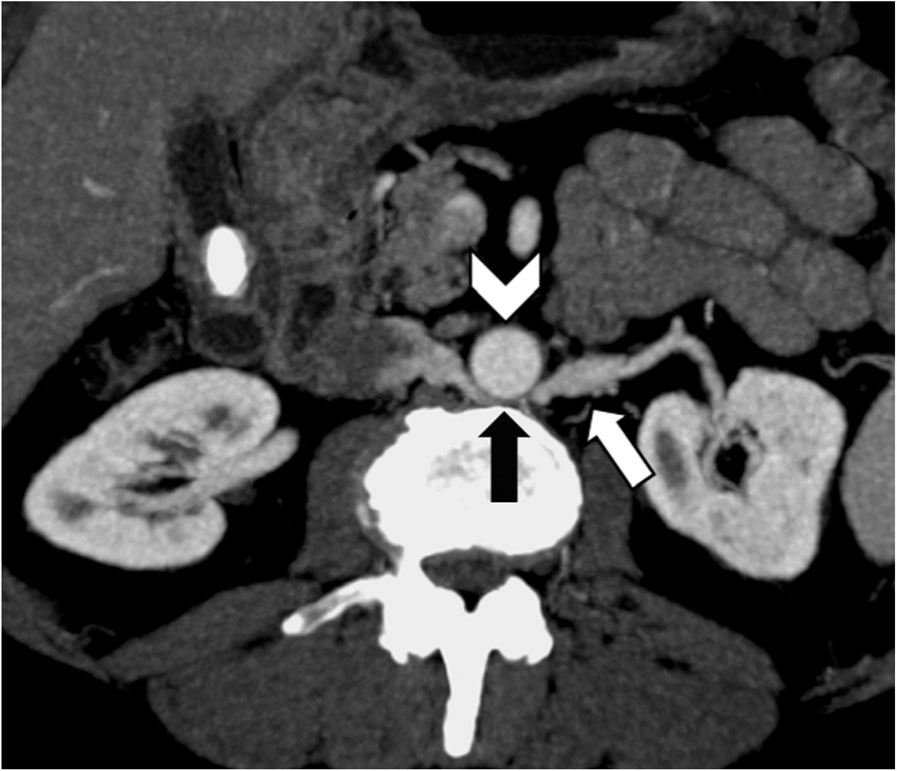
A 78-year-old man with posterior nutcracker syndrome. Axial CT image on the arterial phase shows left renal vein (white arrow) compressed between the aorta (arrowhead) and the vertebral body (black arrow)

**Fig. 7:**
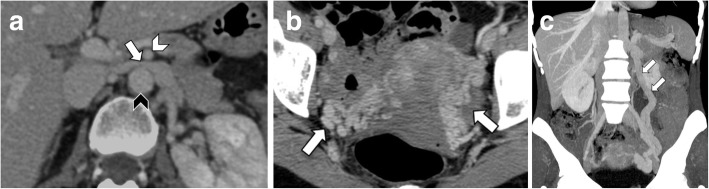
A 50-year-old woman with anterior nutcracker syndrome. **a** Axial contrast-enhanced CT image on the portal venous phase shows the typical “beak sign” (arrow) with left renal vein compression between the aorta (black arrowhead) and superior mesenteric artery (white arrowhead). **b** CT image at the level of the pelvis demonstrates multiple pelvic varices (arrows). **c** Coronal reformatted CT MIP image shows marked dilatation of the left ovarian vein (arrows)

The preaortic or retroaortic course of the LRV and the compression between the abdominal aorta and the SMA can be accurately visualized on CT images acquired on arterial or portal venous phases (Fig. 6) [18]. Axial CT images show the typical “beak sign” of LRV (Fig. 7a), which consists of an abrupt narrowing of the LRV with an acute angle below the aortomesenteric junction [15, 18]. Sagittal CT images allow to calculate the aortomesenteric angle (AMA) between SMA and abdominal aorta, which normally ranges from 38° to 56° [19]. If the AMA decreases to 9–35°, external compression of both the duodenum (SMA syndrome, see below) and left renal vein (NCS) may occur [19, 20]. Indeed, the diagnosis of anterior NCS may be made when AMA is less than 35° [21]. Moreover, the aortomesenteric distance shows a decrease from a normal value of 10–28 mm to 2–8 mm [1, 20]. CT can also depict the resulting gonadal vein dilatation and pelvic varices (Fig. 7), although it does not allow to measure flow velocity and direction [15]. Color Doppler ultrasound may demonstrate a significantly increased peak systolic velocity ratio between the point of compression and the hilar LRV [1, 15]. Despite being more invasive, retrograde venography depicts the level of compression and allows the determination of the reno-caval pressure gradient, which is diagnostic for NCS over 3 mmHg [15, 22].

Management of NCS can be either conservative and observational in patients with mild hematuria and tolerable symptoms or invasive in case of recurrent gross hematuria, severe symptoms or ineffective conservative treatment [15, 23]. Invasive treatments include open surgical and endovascular interventions [24]. Surgical treatment of anterior NCS consists in the transposition of LRV or left gonadal vein in the inferior vena cava, saphenous vein cuff, or vein patch, while anterior reimplantation of retroaortic LRV into the inferior vena cava is performed in posterior NCS [24]. Finally, endovascular LRV stenting may be considered for the treatment of NCS associated with pelvic congestion [15, 25].

## May-Thurner syndrome

**Fig. 8:**
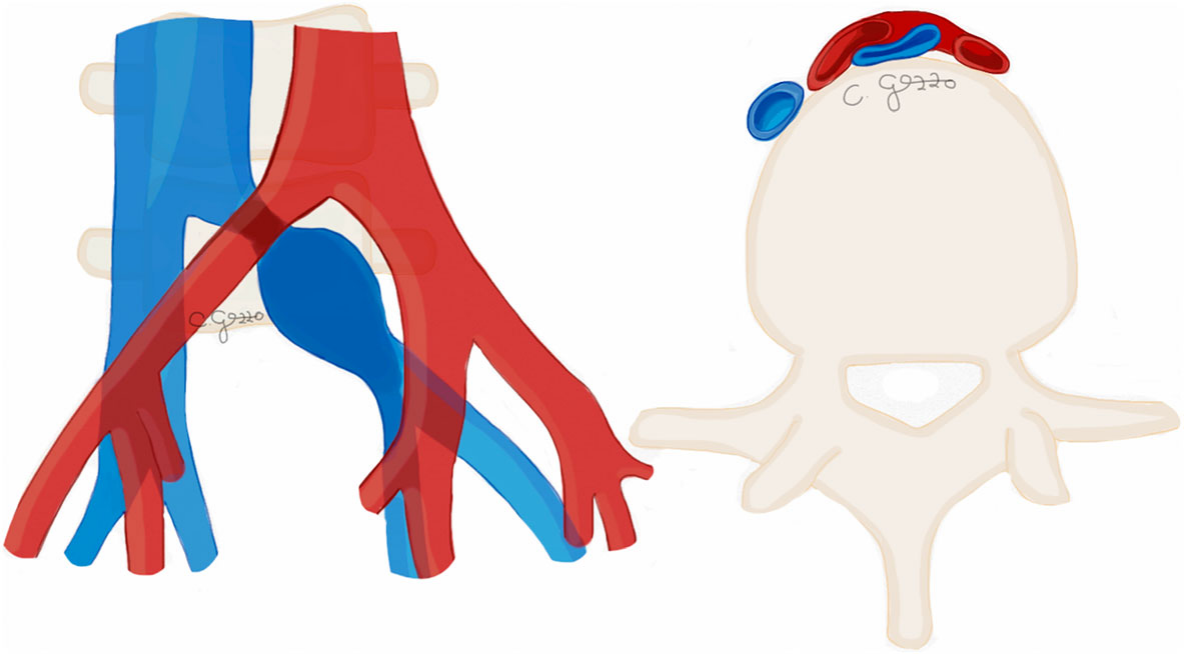
Drawing illustrating the coronal and axial views of May-Thurner syndrome

May-Thurner syndrome (MTS), also described with the eponymous of “Cockett syndrome”, refers to the chronic compression of the left common iliac vein between the overlying right common iliac artery, anteriorly, and the fifth lumbar vertebra, posteriorly (Fig. 8) [2]. Variants of MTS include the right common iliac vein compression by the right common iliac artery or the left iliac vein compressed by the left common iliac artery [26]. MTS is mainly discovered in women between the 2nd and 4th decades of life [1, 14]. The exact prevalence of MTS is unknown, but it is assessed to occur in 2–5% of patients presenting with lower extremity venous disorders [27].

MTS can lead to chronic venous stasis, determining left iliac and femoral veins thrombosis, clinically manifesting with unilateral left lower extremity swelling, edema, varicose veins, venous ulcers, and other potential severe complications, like acute pulmonary embolism or phlegmasia cerulea dolens [1, 2]. This latter is characterized by blue and painful leg, due to massive deep venous thrombosis combined with arterial occlusion determined by concomitant compartment syndrome [28]. Although venous obstruction is commonly caused by extrinsic factors (physical compression of the left common iliac vein), it could also be secondary to intrinsic factors, particularly to endothelial damage due to chronic pulsatile force of the overlying right common iliac artery [1, 29].

**Fig. 9:**
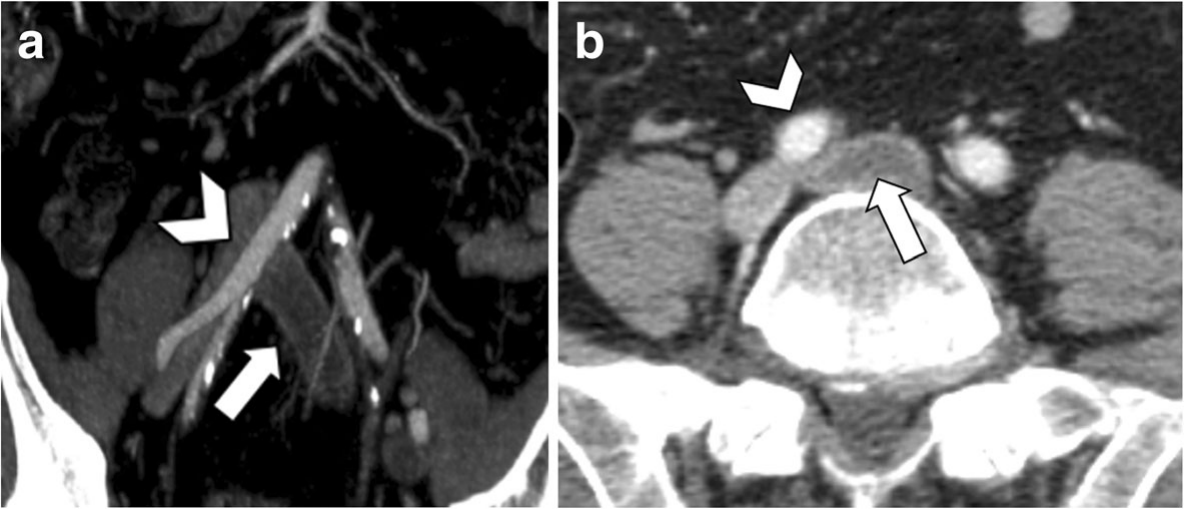
An 85-year-old man with May-Thurner syndrome. Coronal (**a**) and axial (**b**) contrast-enhanced CT images show thrombosed left common iliac vein (arrows) compressed between the right common iliac artery (arrowheads) and fifth lumbar vertebra

Doppler ultrasound may depict the presence of deep venous thrombosis of the left lower limb. As opposed, left iliac vein thrombosis may be challenging to diagnose because this vessel lies deep in the pelvis and may be concealed by gas-filled intestinal loops [1]. Additional imaging with contrast-enhanced CT should be required in the case of suspected MTS [29]. Contrast-enhanced CT images acquired after the intravenous administration of contrast agent on venous and delayed phases are often sufficient to visualize the iliac vein compression and adjacent deep vein thrombosis (Fig. 9). Moreover, CT helps to exclude other conditions such as pelvic masses or lymphadenopathy compressing the left iliac vein or inferior vena cava [1, 5]. Ascending femoral or iliac venography may be performed to confirm the diagnosis in most difficult cases and to measure the pressure gradients, although this is an invasive procedure [14].

Conservative treatments with compression stockings can be sufficient in the absence of deep venous thrombosis. In the case of symptoms, endovascular stent placement is considered the treatment of choice to relieve the mechanical obstruction [1]. Thrombolysis or anticoagulation therapy with compression stockings are only indicated in patients with acute venous thrombosis [1, 14, 30]. An open surgical thrombectomy is exceptional and the only indication is the failure of endovascular therapies [27]. Other surgical options include vascular transposition, venous bypass procedure, and venoplasty with excision of intraluminal bands [30].

## Superior mesenteric artery syndrome

**Fig. 10:**
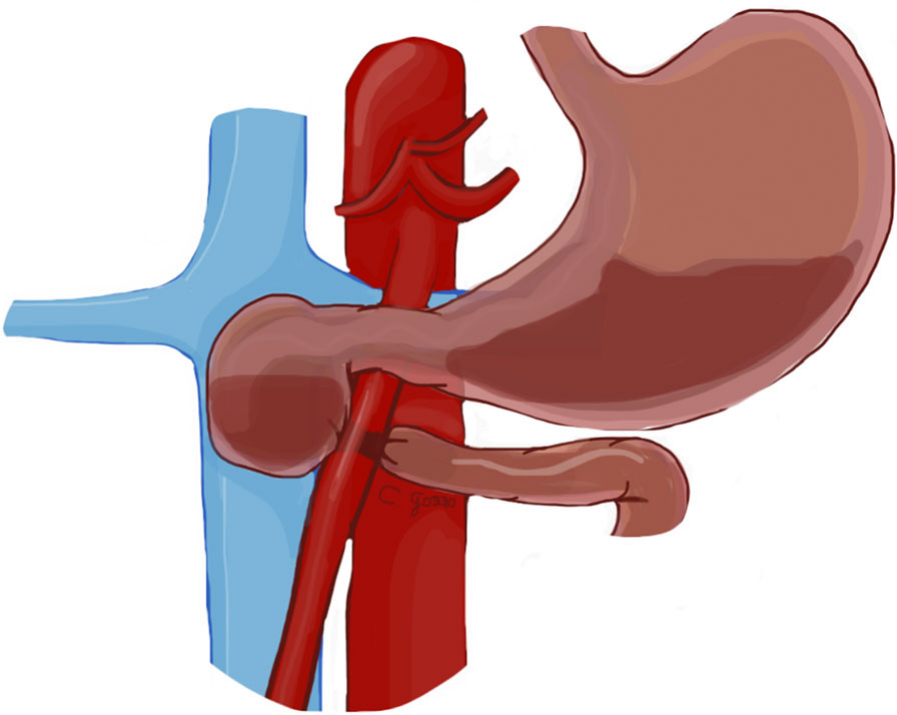
Drawing illustrating the coronal view of superior mesenteric artery syndrome

Superior mesenteric artery syndrome, otherwise known as “Wilkie syndrome”, is caused by a compression of the third portion of the duodenum between the abdominal aorta and the superior mesenteric artery itself, resulting in duodenal obstruction (Fig. 10) [1]. The prevalence of SMA syndrome is around 0.0024–0.03% and it may coexist with anterior NCS due to the common pathogenesis, as detailed above [31]. Congenital or acquired anatomic abnormalities with reduction of aortomesenteric angle or loss of normal perivascular fatty cushion are the most common etiologies of SMA syndrome. A congenital abnormal low origin of the SMA or a high attachment of the angle of Treitz, that displaces the duodenum cranially, may be predisposing conditions [32]. Similarly to NCS, severe weight loss and low body mass index are acquired predisposing factors, due to the loss of the normal mesenteric adipose tissue around the superior mesenteric artery, which normally acts as a fatty cushion [5]. Other acquired abnormalities may be associated with surgery, spinal trauma, burns, eating disorders, neoplastic diseases, and malabsorption states [19, 32].

The clinical signs and symptoms of SMA syndrome include duodenal obstruction manifesting with postprandial abdominal pain, loss of weight, nausea, and vomiting due to the slower gastric emptying. Usually, the SMA syndrome is a diagnosis of exclusion, made when high clinical suspicion is followed by imaging confirmation.

**Fig. 11:**
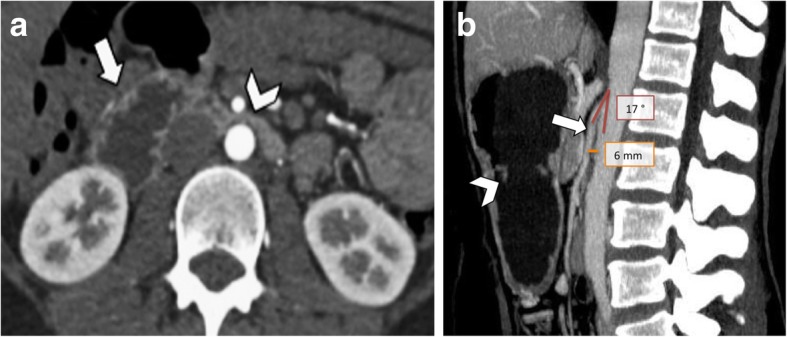
A 48-year-old woman with superior mesenteric artery syndrome. **a** Axial CT image on the arterial phase shows dilatation of the second portion of the duodenum (arrow), due to the compression of the third portion (arrowhead) between the abdominal aorta and the SMA. **b** Sagittal CT image of the abdomen shows gastric dilatation (arrowhead) caused by compression of the third portion of the duodenum between the abdominal aorta and the SMA (arrow). This sagittal reconstruction also allows measurement of aortomesenteric angle (17°) and aortomesenteric distance (6 mm), values diagnostic for mesenteric artery syndrome

The typical findings of SMA syndrome on CT imaging are the compression of the third portion of the duodenum, with upstream severe dilatation of the proximal duodenum and the stomach (Fig. 11a) [1]. As detailed above, sagittal CT images allow to measure accurately the aortomesenteric angle and distance on the arterial phase [19, 20]. CT cut-offs for the diagnosis of SMA syndrome are an AMA lower than 22° and an aortomesenteric distance shorter than 8 mm [5] (Fig. 11b). Differential diagnosis that can be ruled out with contrast-enhanced CT includes other causes of megaduodenum, such as small bowel obstruction, annular pancreas, tumors, inflammatory lesions, aneurysms, or mesenteric ischemia [33, 34].

The management of SMA syndrome is usually conservative and includes decompression through nasogastric tube placement to restore a normal aortomesenteric distance and relieve the obstruction [1]. In the case of failure of conservative management, surgical duodeno-jejunostomy is indicated for patients with severe symptoms [5].

## Ureteropelvic junction obstruction

**Fig. 12:**
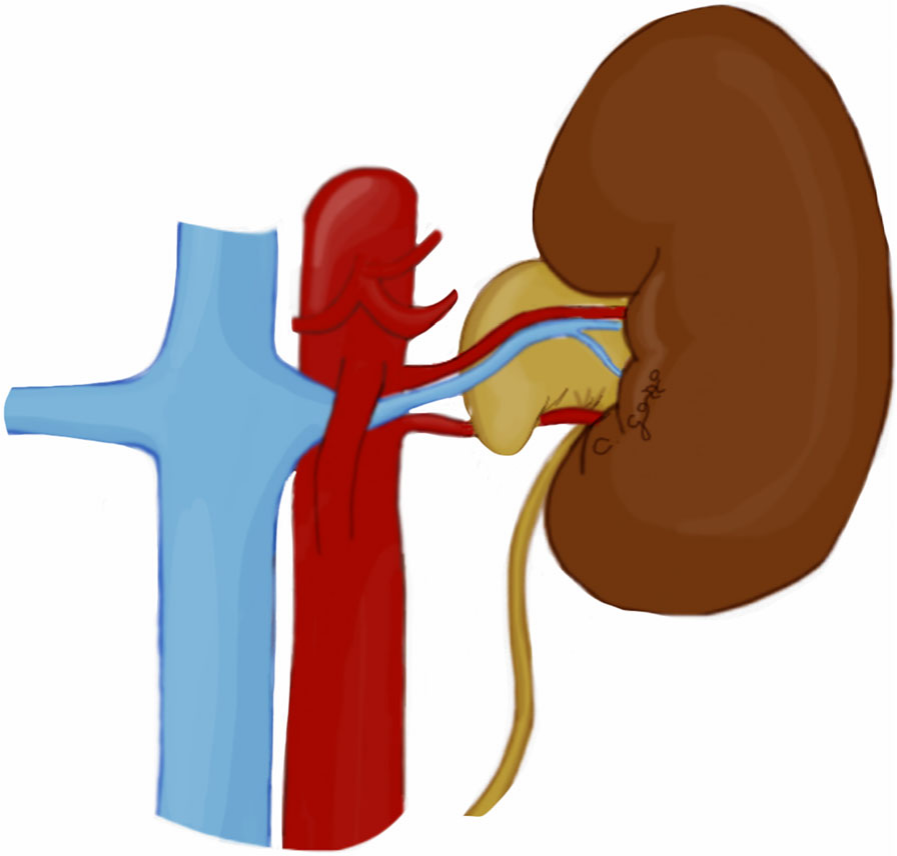
Drawing illustrating the coronal view of ureteropelvic junction obstruction

Ureteropelvic junction obstruction (UPJO) refers to functional or anatomic obstruction of the upper urinary tract at the confluence of the renal pelvis with the upper part of the ureter. Several causes, congenital or acquired, are implicated in UPJO, most commonly “crossing vessels” passing above the ureteric transition point. Particularly, the lower pole segmental renal vessels (artery or vein) may arise from the main renal artery or vein, or branch as accessory vessels directly from the abdominal aorta, iliac artery, or inferior vena cava [1]. Lower-pole segmental artery can bow anteriorly (Fig. 12) or posteriorly over the ureter. Lower pole crossing vessels are found in about 20% of healthy patients, while their incidence is up to 45% in patients with UPJO [35].

UPJO usually remains asymptomatic and it may be incidentally discovered. However, if left untreated, it may manifest with flank pain due to hydronephrosis and can be complicated by hematuria, urolithiasis, urinary tract infections, or pyelonephritis [36]. Secondary UPJO from iatrogenic injuries, inflammation, or neoplastic masses should be considered in the differential diagnosis [36].

**Fig. 13:**
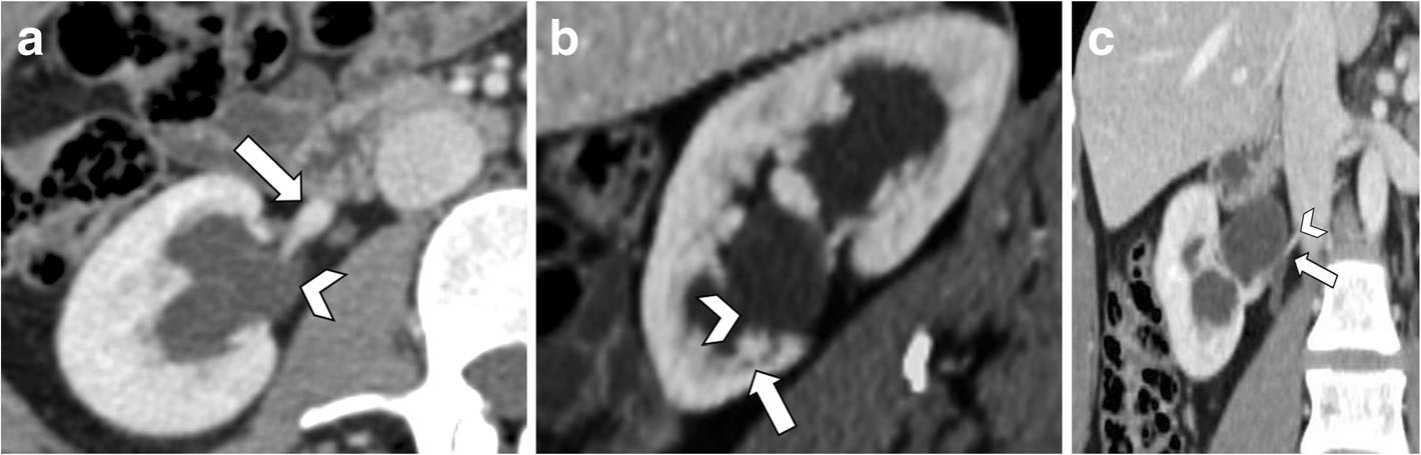
A 31-year-old woman with ureteropelvic junction obstruction. **a** Axial CT image shows pelvic dilatation (arrowhead) caused by ureteropelvic junction compression by lower pole segmental renal artery (arrow). **b** The image reconstructed through an oblique sagittal plane, known as “hilar clock-face view”, obtained orthogonal to the renal hilum, shows the relationship between the lower pole segmental renal artery (arrow) and the ureteropelvic junction (arrowhead). **c** Coronal CT image on the portal venous phase shows the ureteropelvic junction compressed (arrow) by lower pole segmental renal artery originating from the abdominal aorta (arrowhead)

Contrast-enhanced CT allows accurate visualization of the relationship between the crossing vessels and the ureteropelvic junction, as well as the severity of the hydronephrosis. Contrast-enhanced CT should include arterial, portal venous, and excretory phases to identify both arteries and veins as well as their relationship with the renal pelvis or ureter [1]. CT imaging features include dilatation of the renal pelvis with a peculiar inverted “teardrop” appearance, which typically “drapes” over the lower pole segmental artery or vein, while the proximal ureter hooks over the blood vessel (Fig. 13) [1]. Images reconstructed through an oblique sagittal plane, known as “hilar clock-face view”, obtained orthogonal to the renal hilum, can better highlight the radial spread of the renal vessels and their relationship to the ureter (Fig. 13b) [36]. The diagnosis of UPJO is particularly relevant for surgical planning since renal arteries are terminal vessels which must be preserved during surgery. Preoperative CT detection of crossing vessels may avoid their accidental iatrogenic injury [1].

Treatment depends on clinical symptoms, the grade of hydronephrosis, and decline in renal function. Surgical treatment of UPJO includes endopyelotomy, pyeloplasty, and vessel transposition [1, 37]. Endopyelotomy could be carried out in the absence of crossing vessels, whereas laparoscopic dismembered pyeloplasty is the treatment of choice if crossing vessels are present [1]. In some patients, vessel transposition without dismembered pyeloplasty may be curative [37].

## Ureteral vascular compression syndromes

**Fig. 14:**
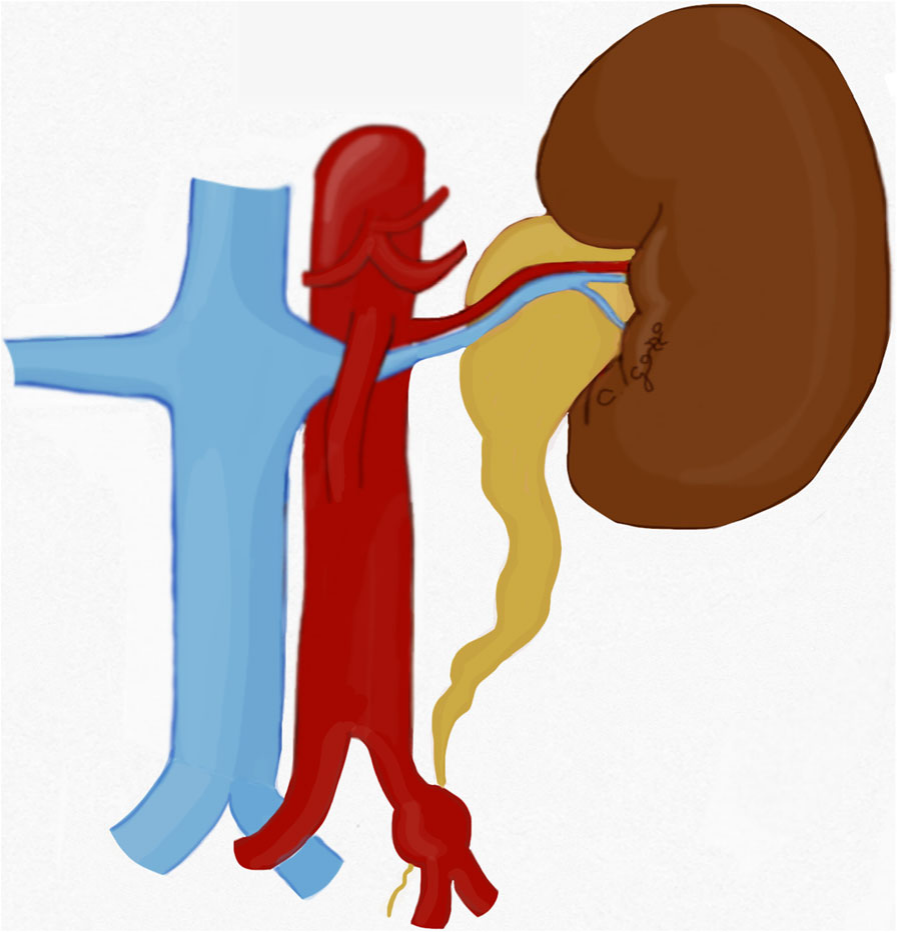
Drawing illustrating the coronal view of ureteral obstruction by common iliac artery aneurysm

**Fig. 15:**
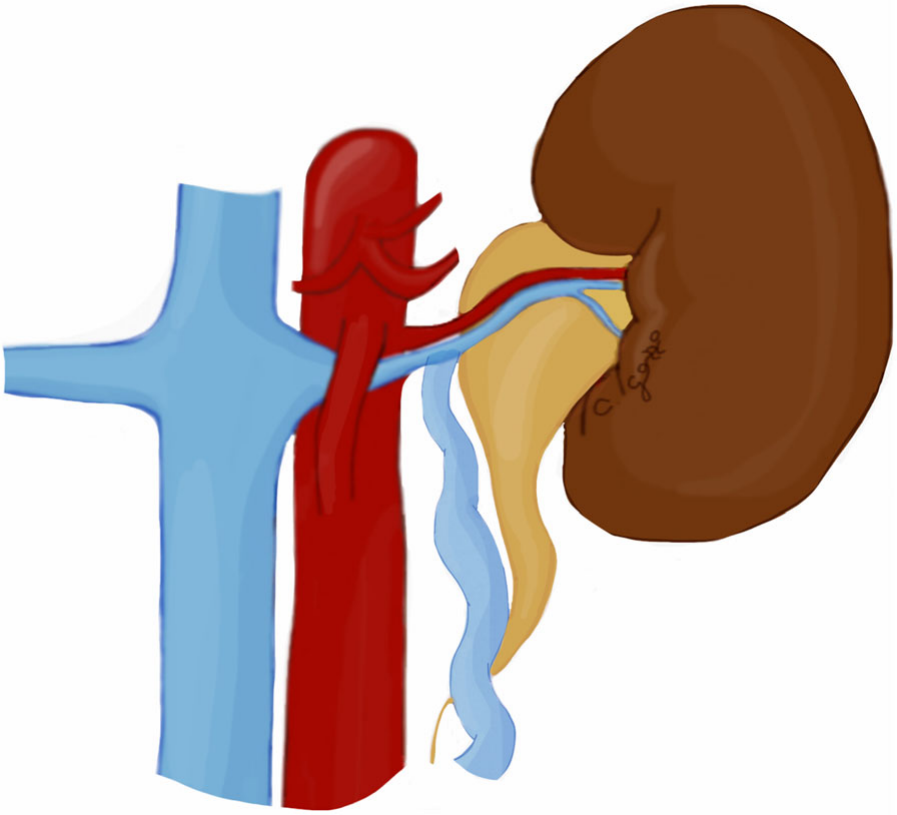
Drawing illustrating the coronal view of left ovarian vein syndrome

Obstructive uropathy by vascular compression may also appear at the point of attachment of the ureter with retroperitoneal vessels, such as the ovarian vein, more infrequently testicular vein or common iliac artery. The ureter may even result in compressed by adjacent common iliac artery aneurysm (Fig. 14), especially inflammatory [38] or mycotic aneurysms [39]. The hydroureteronephrosis secondary to a compression by a dilated or aberrant ovarian vein is known as “ovarian vein syndrome” (OVS) (Fig. 15) [1]. OVS is most commonly located on the right side and usually affects thin women [40]. It occurs most frequently during pregnancy due to the hormonal changes with ovarian vein dilatation, valvular incompetence, and decreased muscular ureteral tone [40]. OVS should be differentiated from pelvic congestion syndrome, which consists of the dilatation of the entire anastomotic pathway of the pelvic venous system (ovarian, uterine, iliac), frequently with caudal extension to the lower extremity veins [41].

Obstruction from ureteral compression can be asymptomatic or clinically manifest with flank pain, hematuria, or pyelonephritis [1].

**Fig. 16:**
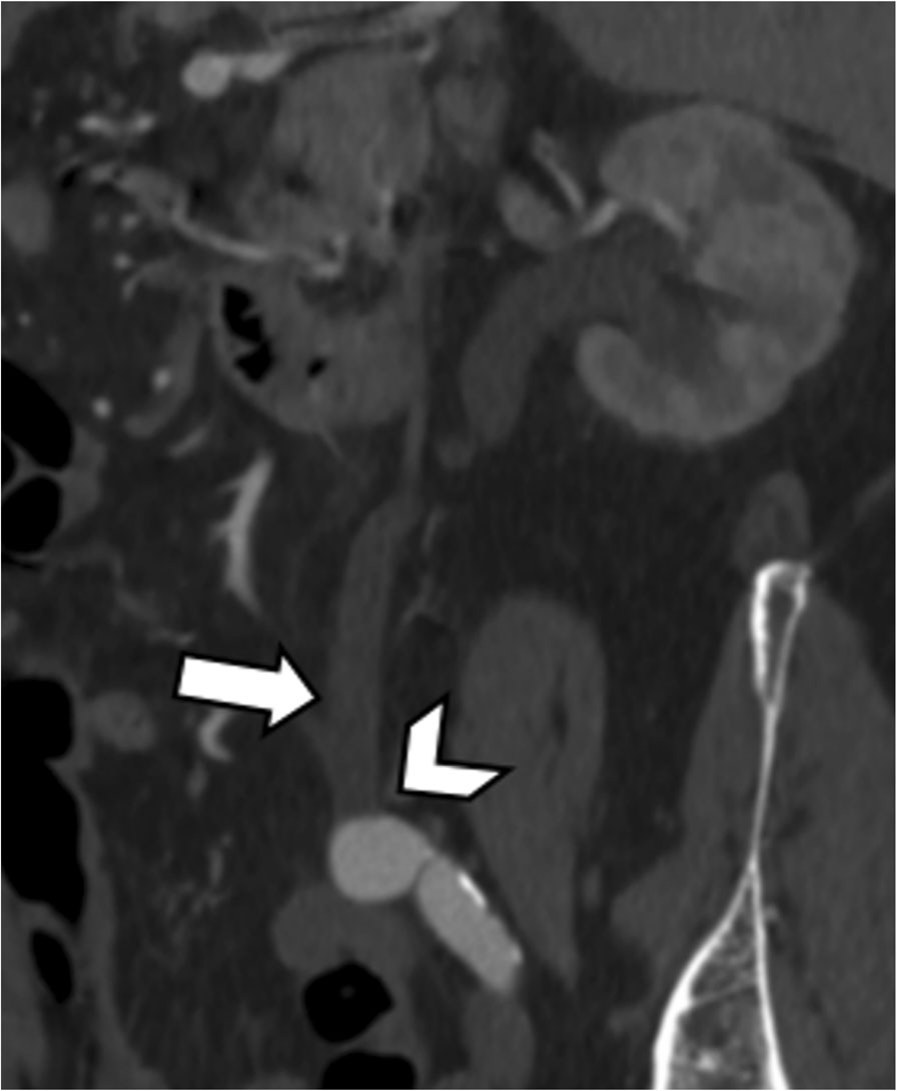
An 83-year-old man with ureteral vascular compression. Sagittal CT image on arterial phase shows right hydroureteronephrosis (arrow) secondary to compression by a saccular aneurysm (arrowhead) of the adjacent right common iliac artery

**Fig. 17:**
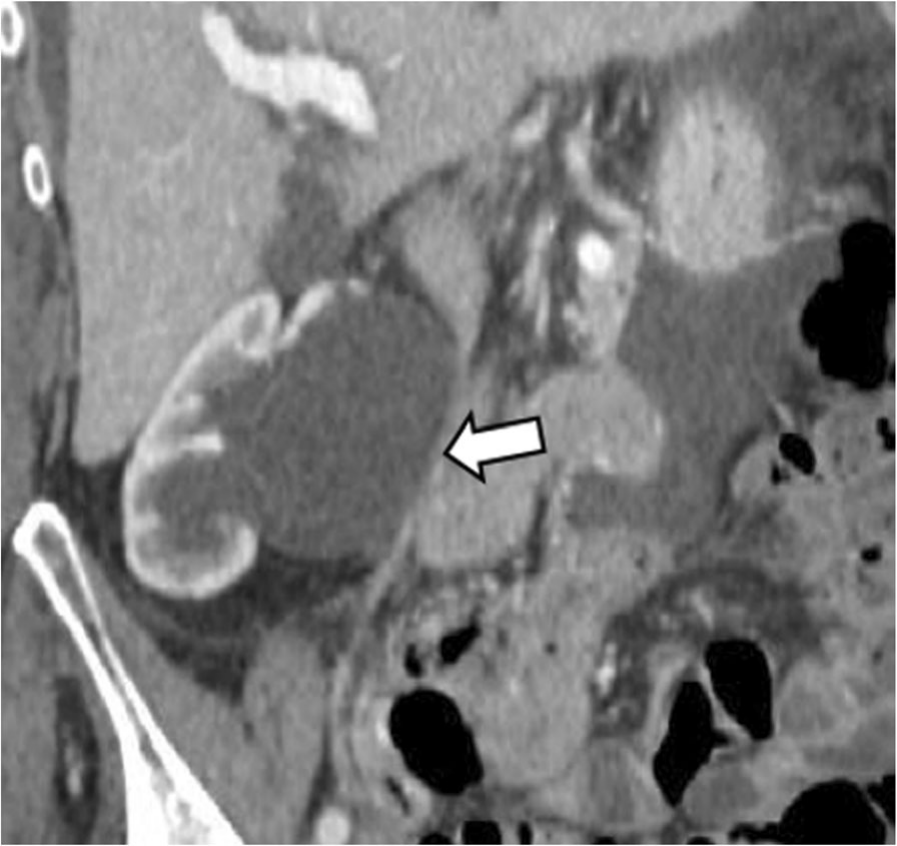
An 84-year-old woman with upper ureteral vascular compression. Coronal enhanced CT image shows the proximal ureter (arrow) compressed by a dilated ovarian vein

Contrast-enhanced CT should be acquired in both arterial, venous, and excretory phases for the correct evaluation of the ureter and adjacent vessels. The excretory phase images allow the visualization of ureteral compression by crossing vessels, with associated hydronephrosis and proximal dilatation of the ureter above the level of obstruction, while the caliber of the distal ureter is normal. These findings are better depicted on coronal images. However, the presence of ureter obstruction and hydronephrosis is not sufficient to make the diagnosis of ureteral vascular compression syndrome. Contrast-enhanced CT images on arterial or venous phase need to depict the presence of a common iliac artery aneurysm at the level of the iliac vessels (Fig. 16**)** or a dilated ovarian vein (Fig. 17) causing ureteral obstruction and exclude other causes of obstruction such as urinary calculi or tumoral strictures.

In symptomatic patients, surgical intervention to relieve the obstruction includes laparoscopic uretero-ureterostomy, in case of retrocaval ureters, and ovarian vein ligation [1]. Transcatheter ovarian vein embolization can be a minimally invasive option in OVS resistant to medical therapy [41].

## Portal biliopathy

**Fig. 18:**
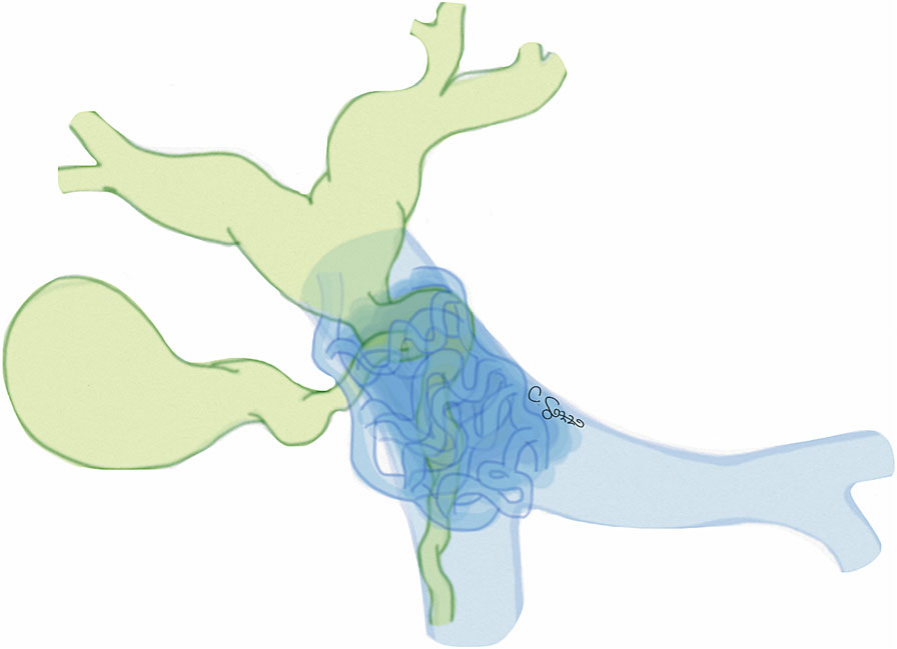
Drawing illustrating the coronal view of portal biliopathy

Portal biliopathy is defined as an abnormal dilatation of the extra- and/or intrahepatic biliary ducts, as well as the cystic duct and gallbladder, due to the presence of chronically thrombosed portal system evolved into “portal cavernoma” [42–44]. Portal cavernoma (Fig. 18) is the cavernous transformation of the venous drainage of the common bile duct formed by the paracholedochal veins (plexus of Petren) and the epicholedochal veins (plexus of Saint), located along the ductal wall [45]. There are two mechanisms responsible for portal biliopathy: an outer mechanical compression of the biliary system by portal cavernous transformation and/or peribiliary fibrosis secondary to inflammatory or ischemic changes caused by thrombosis of small veins in the bile duct walls [44, 46]. Indeed, portal biliopathy has been classified into three types: varicoid, fibrotic, and mixed. The varicoid type is the compression and distortion of the bile duct caused by large collateral veins (paracholedochal veins). The fibrotic type shows the tricking and narrowing of the bile duct, resulting from compression of smaller intramural collateral veins (epicholedochal veins) [46]. Chandra et al. [45] proposed an additional classification system according to the location of biliary obstruction with various combinations of extrahepatic, intrahepatic, unilateral, or bilateral intrahepatic ducts involvement.

About 70–100% of patients with radiological evidence of portal biliopathy are initially asymptomatic [44]. Portal biliopathy becomes clinically manifest only in a minority of patients with chronic cholestasis and jaundice [42, 43, 46]. Overall, 4–10% patients may develop complications like choledocholithiasis, cholangitis, and secondary biliary cirrhosis [47].

**Fig. 19:**
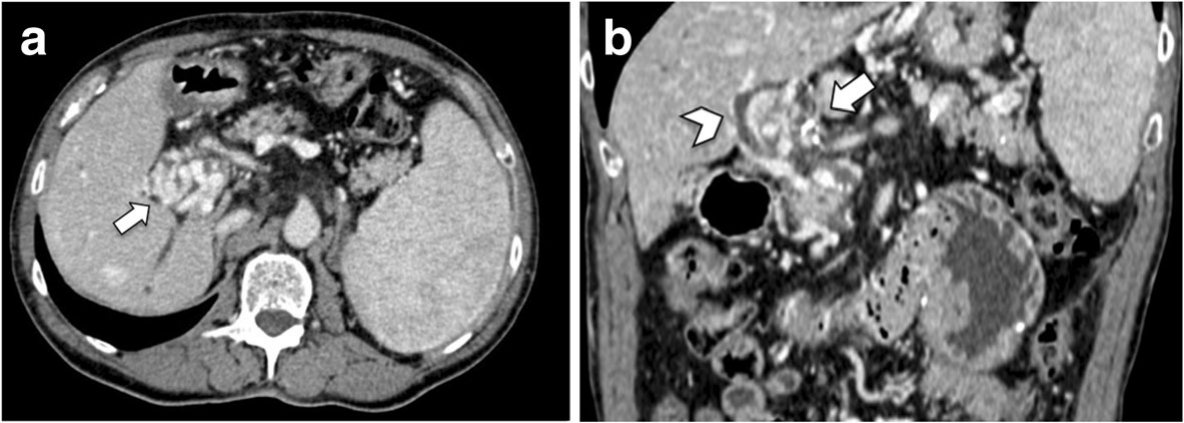
A 63-year-old woman with portal biliopathy. **a** Axial enhanced CT image shows the cavernous transformation of the portal vein (arrow). **b** Coronal CT image demonstrates biliary ducts dilatation (arrowhead) due to compression by portal cavernoma (arrow)

Ultrasound may demonstrate the intra- and extra-hepatic bile duct dilatation and the imaging findings of portal cavernous transformation [46], including multiple anechoic tubular structures with vascular flow at color Doppler, corresponding to paracholedochal collateral veins in hepatoduodenal ligament and porta hepatis, and increased flow in hepatic artery as a compensatory mechanism [46]. Contrast-enhanced CT (Fig. 19) is mainly performed to exclude other causes of biliary dilatation since it can mimic malignant strictures of the distal bile duct from pancreatic adenocarcinoma or biliary neoplasms [43]. The most common imaging finding of portal biliopathy, besides the portal cavernoma, is the acute angulation of the common bile duct forming a “kinking”, which can be considered the consequence of the extrinsic compression by the dilated paracholedochal veins [48]. When MRI with cholangiography is performed, it may demonstrate a “scalloping” or “wavy” delineation of the extrahepatic biliary ducts secondary to indentations by the dilated peribiliary veins [48].

Portal biliopathy does not require specific treatment in asymptomatic patients. Symptomatic subjects may be approached with interventional radiology or surgical management [43]. Interventional radiology plays a role in biliary drainage, through the placement of nasobiliary or biliary stent, or in portal vein recanalization and transjugular intrahepatic portosystemic shunt (TIPS) placement in selected patients with chronic portal vein thrombosis [49, 50]. In the case of persistent biliary obstruction, a surgical decompression may be performed with hepaticojejunostomy or choledochoduodenostomy [43].

## Conclusions

Abdominopelvic vascular compression syndromes may cause a wide spectrum of atypical abdominal symptoms or acute complications. Anatomical findings predisposing to these compression syndromes may be discovered even in asymptomatic subjects who undergo imaging for unrelated indications. In patients with suspected vascular compression syndrome, the combination of clinical symptoms, appropriate imaging modalities, and knowledge of typical imaging features is important for their accurate diagnosis and to guide the appropriate management. Particularly, contrast-enhanced CT may be performed with different protocols according to the specific syndrome in order to maximize the visibility of involved vessels and allow the identification of typical imaging features and possible complications.

## Abbreviations

AMA: Aortomesenteric angle
CT: Computed tomography
LRV: Left renal vein
MALS: Median arcuate ligament syndrome
MRI: Magnetic resonance imaging
MTS: May-Thurner syndrome
NCS: Nutcracker syndrome
OVS: Ovarian vein syndrome
SMA: Superior mesenteric artery
TIPS: Transjugular intrahepatic portosystemic shunt
UPJO: Ureteropelvic junction obstruction
